# Is There a Correlation Between Preoperative HbA1c Change, Long-Term Weight Loss and Glycaemic Control in Patients With Type 2 Diabetes Undergoing Metabolic Surgery?

**DOI:** 10.7759/cureus.70921

**Published:** 2024-10-06

**Authors:** Jennifer Tempany, Abdulmajid Ali, Andrew Collier

**Affiliations:** 1 Bariatric Surgery Unit, University Hospital Ayr, Ayr, GBR; 2 School of Health and Life Sciences, University of the West of Scotland, Glasgow, GBR; 3 School of Health and Life Sciences, Glasgow Caledonian University, Glasgow, GBR

**Keywords:** glycaemic control, long-term weight loss, metabolic surgery, obesity, type 2 diabetes

## Abstract

Introduction

Optimisation of patients with type 2 diabetes mellitus (T2DM) prior to metabolic surgery aims to achieve tight glycaemic control by the time of surgery. Little is known about the influence of altering preoperative glycated haemoglobin (HbA1c) on postoperative weight loss and glycaemic control. The aim of this study was to determine whether a change in HbA1c during the preoperative period correlated with long-term weight maintenance and HbA1c in patients undergoing metabolic surgery. The quantity of glucose-lowering medication used prior to and following surgery was also examined.

Methods

A retrospective analysis was conducted on patients with T2DM who underwent metabolic surgery between 2013 and 2017. Preoperative HbA1c change was measured as a change in glycaemic control during the one-year pre-surgery. The primary outcomes were % excess weight loss (EWL) and HbA1c at five-year post-surgery. Secondary outcomes were % EWL and HbA1c at one-year post-surgery and the use of glucose-lowering medications post-surgery. The Pearson correlation coefficient *(r)* was used to determine the relationship between the pre-surgery HbA1c change and postoperative % EWL and HbA1c. A chi-squared test was used to calculate the statistical impact of changes in medication use post-surgery.

Results

Sixty-nine patients with complete data were included in the study. The mean change in HbA1cin the one-year pre-surgery, the one-year post-surgery and five-year post-surgery was -0.9% (1.5), -0.7% (1.2) and 0% (0 1.8), respectively. A change in HbA1cin the one-year pre-surgery did not correlate with % EWL at one-year and five-year post-surgery or with HbA1cat one-year and five-year post-surgery. At one-year and five-year post-surgery, there was a significant decrease in the proportion of patients requiring glucose-lowering medications compared to patient use prior to surgery (p < 0.001).

Conclusion

This study demonstrated a significant reduction in the proportion of glucose-lowering medication required long-term following metabolic surgery. Altering preoperative glycaemic control was not associated with long-term weight maintenance or glycaemic control.

## Introduction

Global trends in overweight and obesity are rising, and it is estimated that worldwide adult obesity rates have nearly tripled since 1975. Obesity is closely linked to the development of type 2 diabetes mellitus (T2DM), and in 2018, the World Health Organization estimated that 90% of adults with T2DM are overweight or obese [1]. Metabolic surgery has been demonstrated as an effective and sustainable method for managing obesity as well as T2DM and has been shown to improve life expectancy in patients living with obesity [2,3]. Postoperatively, metabolic alterations reduce adiposity, leading to improvements in insulin sensitivity and glucose homeostasis [4]. Glycaemic control improves immediately, and often, diabetes remission is achieved [5,6]. When remission is not achieved, a reduction of glucose-lowering medication has been shown to occur [7]. Multiple studies have alluded to preoperative glycated haemoglobin (HbA1c) as a predictor of postoperative outcomes in metabolic surgery. Patients with poorer glycaemic control at the time of surgery have demonstrated worse outcomes, such as lower rates of diabetes remission and less postoperative weight loss [8,9].

International guidelines published in 2019 suggest patients should aim to achieve an HbA1c between 6.5% and 7.0% prior to surgery through engagement in a comprehensive care plan [10]. A preoperative care plan for patients living with diabetes is centred around intensive lifestyle modifications, including health-focused dietary changes and physical exercise regimes, with this intervention frequently being instigated in the form of a preoperative patient-focused education program. Glucose-lowering medications are used in conjunction with lifestyle interventions when indicated to optimise preoperative HbA1c [11]. Prior focus has been centred on how preoperative lifestyle interventions impact on postoperative weight loss outcomes [12,13]. There is, however, a scarcity of literature addressing the influence of altering glycaemic control during this preoperative period on postoperative long-term weight loss and glycaemic control. Two studies have looked at the impact of optimising glycaemic control prior to metabolic surgery on postoperative glycaemic changes, and the outcomes show conflicting results.

The first study looked at the impact of glucose optimisation in the six months prior to Roux-en-Y-gastric bypass (RYGB) and found that patients who had a decrease in HbA1c prior to surgery by 1% were 68% more likely to achieve diabetes remission at one-year following surgery [14]. The second study, the GLUCOSURG-pre randomised control trial, compared the impact of intensive glucose optimisation in the three months prior to undergoing an RYGB in patients with an HbA1c of ≥8.5% with patients receiving standard of care. The primary outcome was HbA1c at one-year post-surgery, and the results demonstrated no statistical difference in HbA1c between the two groups [15]. No studies to date, to the best of the authors’ knowledge, have looked at the impact of altering preoperative HbA1c on long-term weight maintenance in metabolic surgery.

Subsequently, this study sought to explore whether a change in HbA1c during the one-year preoperative period correlated with long-term postoperative maintenance of weight loss and HbA1c in patients undergoing laparoscopic RYGB (L-RYGB) or laparoscopic sleeve gastrectomy (LSG). This study also considered weight loss and glycaemic control at one-year post-surgery and the proportion of glucose-lowering medications utilised long term. In the author’s centre, patients engaged in an intensive preoperative education program centred around optimising lifestyle changes prior to undergoing surgery.

## Materials and methods

Patient data were reviewed from the NHS Ayrshire & Arran bariatric database and the Scottish Care Information Diabetes platform. All patients with T2DM who underwent L-RYGB or LSG were retrospectively identified between 2013 and 2017. Patients requiring glucose-lowering medication, insulin or diet alone preoperatively were included in this study. Glucose-lowering medications were defined as metformin, glucagon-like peptide 1 (GLP-1) agonists, sulfonylureas, thiazolidinediones, meglitinides and dipeptidyl peptidase 4 (DPP-4) inhibitors and sodium-glucose cotransporter-2 (SGLT2) inhibitors. 

The difference in % HbA1c in the one-year pre-surgery was used as a measurement of preoperative HbA1c change due to patient enrolment occurring at this time in NHS Ayrshire & Arran Bariatric Centre. The difference in HbA1c was calculated by subtracting the % HbA1c one-year pre-surgery from the % HbA1c at the time of surgery. The % HbA1c changes at one- and five-year post-surgery were calculated by subtracting the % HbA1c at the given postoperative time point from the % HbA1c at the time of surgery. Data retrieved from the Scottish Care Information Diabetes platform were obtained during patient enrollment plus review appointments, and HbA1c results were included, and samples were collected within two months of the referenced time point.

As an indicator of weight maintenance and glycaemic control, % EWL and HbA1c at five-year post-surgery were used as the primary outcomes, respectively. Secondary outcomes were % EWL and % HbA1c at one-year post-surgery and the use of glucose-lowering medications post-surgery. The % EWL was calculated as ((preoperative weight (kg) − follow-up weight (kg)) / (preoperative excess weight (kg))) × 100, where the preoperative excess weight was calculated as (preoperative weight (kg) − ideal body weight (kg)) and where the ideal body weight was based on BMI 25kg/m2. From the remaining data, all patients with documented % HbA1c and accurate weights (kg) at the given time points were included in the analysis. The study also compared the medication intake at the preoperative time, one-year post-operation, and five-year post-operation to determine the effect of medication on long-term glycaemic control.

The Pearson correlation coefficient (r) was carried out to determine the correlation between preoperative HbA1c change and the % EWL, as well as % HbA1c at one- and five-year post-surgery. A p-value of <0.05 was considered statistically significant. In the results, given multiple p-values were large, suggesting no correlation between the variables, a linear regression model using the same t-test in the form of a regression model: Y = a + Bx was used to further support the lack of correlation between preoperative HbA1c change and postoperative variables. The p-value was the same p-value calculated in the Pearson method. R2 was the square of the correlation between the two variables in the linear regression. Chi-squared McNemar's test was used to determine the statistical significance of glucose-lowering medication changes post-surgery. All data analysis was performed with Microsoft Excel 2023 (Microsoft Corp., Redmond, WA). 

Pre-surgery intervention

Patients participated in a preoperative six-session educational programme lasting approximately three months, focusing on dietary modifications and physical activity interventions. Patients received multidisciplinary input from a bariatric dietician, psychologist, endocrinologist, metabolic surgeon and bariatric nurse specialist. In NHS Ayrshire & Arran, patients aimed to achieve a target HbA1c of ≤7.5% pre-surgery.

## Results

There were 89 patients with T2DM who underwent metabolic surgery between 2013 and 2017. Twenty patients had incomplete data; therefore, 69 patients were included in the follow-up study. The mean age at surgery was 49.1 years old, and the mean BMI was 41.9 kg/m^2^. The patient’s age range was between 28 and 62, and there were more females (56.5%) than males (43.5%). Patients who underwent LSG amounted to 55.1%, while 44.9% of patients underwent L-RYBG. The baseline characteristics are outlined in Table 1. 

**Table 1:** Baseline characteristics of patients at surgery, (n = 69) IQR: interquartile range, L-RYBG: laparoscopic Roux-en-Y gastric bypass, LSG: laparoscopic sleeve gastrectomy

Characteristic		At surgery
Female, n (%)		39 (56.5)
Age (years), mean ± SD		49.1 ± 8.7
Age range (years)		28-62
Duration of diabetes (years), median (IQR)		3.5 (2-7.75)
Type of surgery	L-RYGB, n (%)	31 (44.9)
	LSG, n (%)	38 (55.1)

The average HbA1c one-year pre-surgery, at the time of surgery, one-year post-surgery and five-year post-surgery was 7.5% (±1.6), 6.6% (±1.1), 5.9% (±1.0) and 6.6% (±0.2), respectively (Table 2). The average change in HbA1c during the one-year preoperative period, from the time of surgery to the one-year postoperative period and from the time of surgery to the five-year postoperative period was -0.9% (±1.5), -0.7% (±1.2) and 0% (±1.8), respectively. In terms of glycaemic control in the postoperative period, 16 patients had a deterioration, five patients had no change, and 48 patients achieved an improvement.

**Table 2:** The change in weight (kg), BMI (kg/m2), HbA1c (%) and % EWL in the postoperative period BMI: body mass index, EWL: excess weight loss, HbA1c: glycated haemoglobin

Characteristic	At surgery	One-year post-surgery	Five-year post-surgery
Weight (kg), mean ± SD	119.4 ± 21.7	94.5 ± 19.6	102.1 ± 2.3
BMI (kg/m^2^), mean ± SD	41.9 ± 6.2	33.0 ± 5.3	36.0 ± 0.7
HbA1c (%), mean ± SD	6.6 ± 1.1	5.9 ± 1.0	6.6 ± 0.2
% EWL, mean ± SD	-	53.7 ± 27.0	36.1 ± 3.4

**Figure 1:**
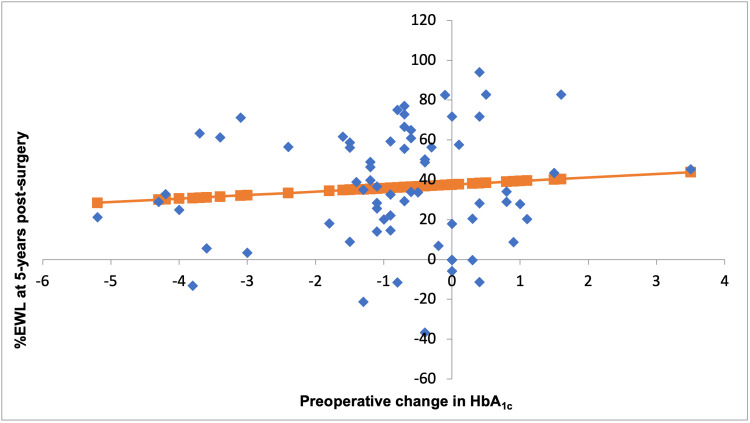
The correlation between the preoperative change in HbA1c (%) and % EWL at five-year post-surgery (p = 0.44) EWL: excess weight loss, HbA1c: glycated haemoglobin

**Figure 2:**
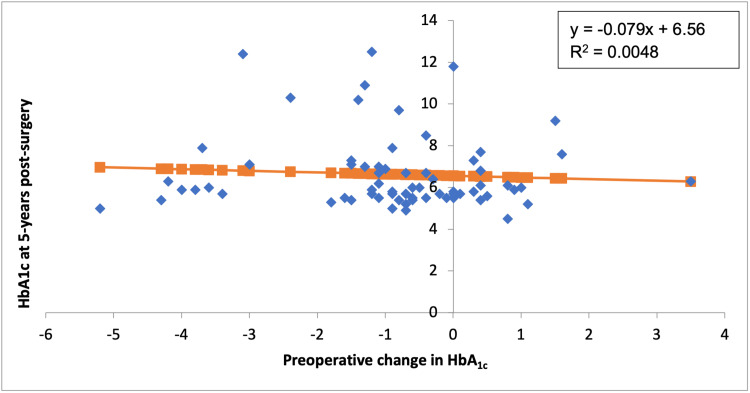
The correlation between the preoperative changes in % HbA1c and % HbA1c at five-year post-surgery (p = 0.57) HbA1c: glycated haemoglobin

A change in HbA1c in the one-year pre-surgery did not correlate with % EWL at five-year post-surgery (Figure 1). At five-year post-surgery, there was a weak positive relationship between the change in preoperative HbA1c and % EWL (p = 0.44). A change in preoperative HbA1c did not correlate with HbA1c at five-year post-surgery (Figure 2). At five-year post-surgery, there was a weak inversely proportional relationship between the change in HbA1c and HbA1c at five-year post-surgery (p = 0.57).

**Figure 3:**
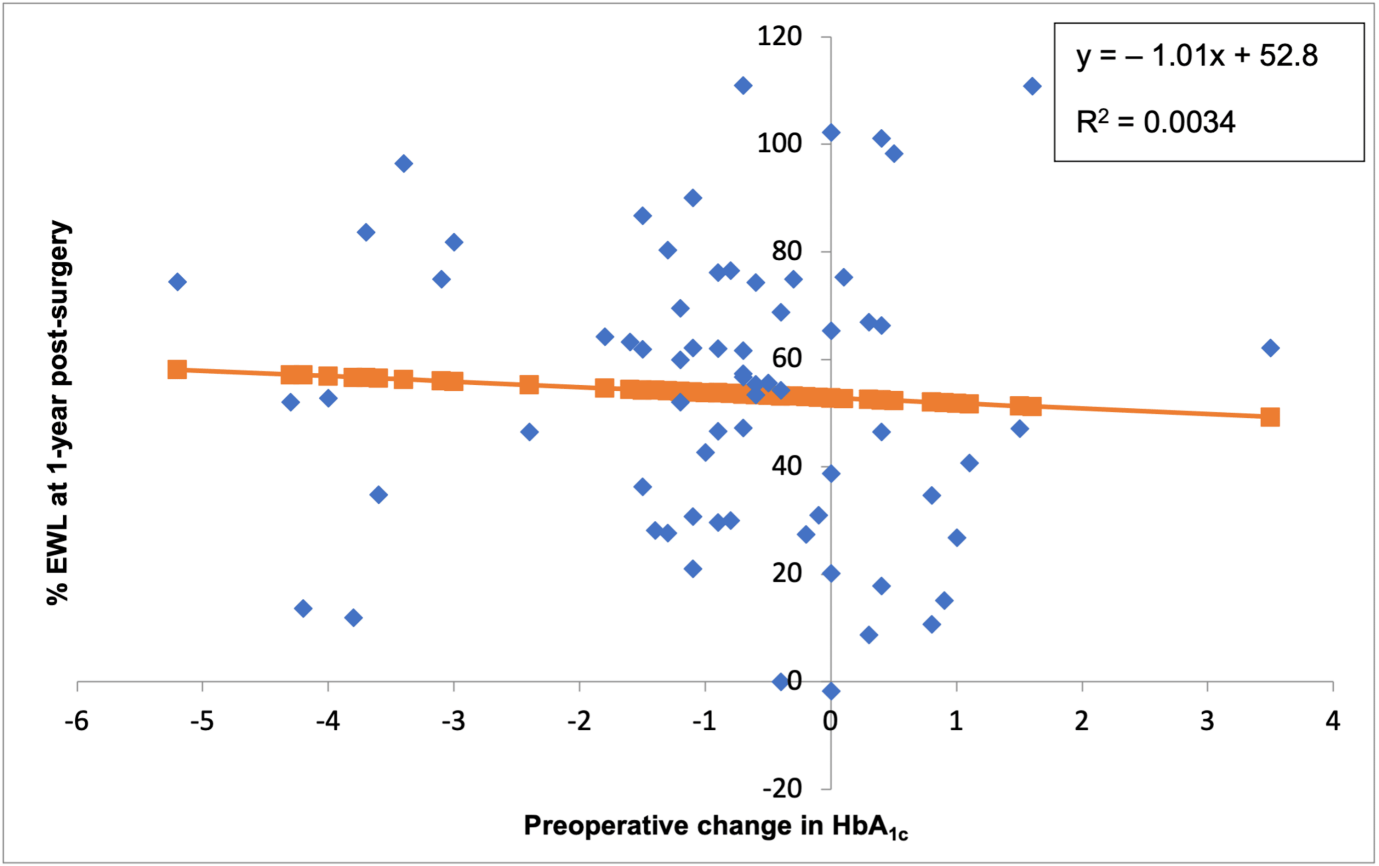
The correlation between preoperative changes in HbA1c (%) and % EWL at one-year post-surgery (p = 0.64) EWL: excess weight loss, HbA1c: glycated haemoglobin

**Figure 4:**
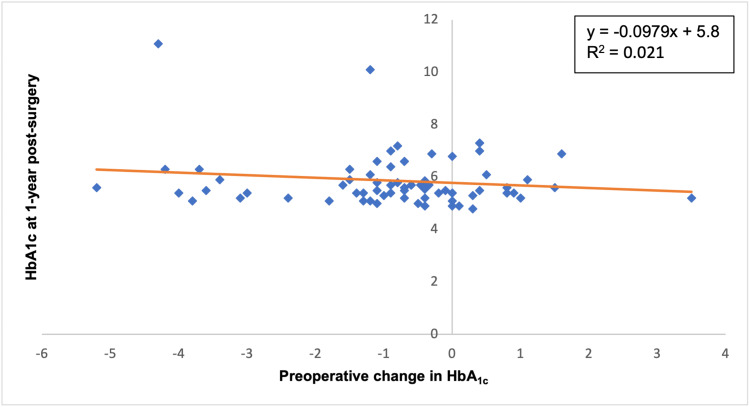
The correlation between the preoperative changes in % HbA1c and % HbA1c at one-year post-surgery (p = 0.24) HbA1c: glycated haemoglobin

A change in HbA1c preoperatively did not correlate with % EWL at one-year post-surgery (Figure 3). At one-year post-surgery, there was a weak inversely proportional relationship between change in HbA1c and % EWL (p = 0.64). A change in HbA1c preoperatively did not correlate with HbA1c at one-year post-surgery (Figure 4). At one-year post-surgery, there was a weak inversely proportional relationship between a preoperative HbA1c change and HbA1c at one-year post-surgery (p = 0.24).

The proportion of patients requiring glucose-lowering medications at the time of surgery, one-year post-surgery and five-year post-surgery was 82.6%, 13% and 33.3%, respectively (Table 3). The decrease in oral glycaemic medication use at one-year and five-year post-surgery compared to patient use at the time of surgery was statistically significant (p < 0.001).

**Table 3:** The proportion of glucose-lowering medication use at surgery, one- and five-year post-surgery (n = 69)

	At surgery	One-year post-surgery	Five-year post-surgery
Patients requiring glucose-lowering medications, n (%)	57 (82.6)	9 (13.0)	23 (33.3)
Reduction in glucose-lowering medication use, n	48 (p < 0.001)		-
	-	34 (p < 0.001)	
Proportion of glucose-lowering medications required, n (%)			
No medication	12 (17.4)	60 (87.0)	46 (66.7)
One oral glucose-lowering medication	25 (36.2)	5 (7.2)	11 (15.9)
Two or more oral glucose-lowering medications	26 (37.7)	3 (4.4)	10 (14.5)
Insulin	6 (8.7)	1 (1.4)	2 (2.9)

## Discussion

This study did not demonstrate a correlation between pre-surgical change in HbA1c and long-term weight maintenance or HbA1c. A significant reduction in the number of glucose-lowering medications required by patients at one-year and five-year post-surgery compared to those at the time of surgery was demonstrated (p < 0.001), with patients achieving an identical average HbA1c five-year post-surgery in comparison to the time of surgery (6.6%).

The significant reduction in glucose-lowering medications five-year post-surgery emphasises the potent metabolic effects of weight loss on glucose homeostasis. Improvements in metabolic control, however, appear to be independent of weight loss in the early postoperative period, with mechanisms being linked to alterations in insulin sensitivity, incretin secretion, improvements in beta cell function and peripheral insulin sensitivity [4,16]. For patients, changes in hunger, satiety, and food preferences may occur, which also aid postoperative weight loss [17]. As patients continue to reduce their weight and glycaemic control improves, glucose-lowering medications can be carefully reduced or withdrawn with multidisciplinary team involvement. A reduction in polypharmacy can be of substantial benefit to patients as multi-drug regimens can be high amongst patients with T2DM due to the aim of achieving disease control and treating associated comorbidities [18]. Polypharmacy has also been associated with increased risk of hospitalisation, adverse drug reactions, drug interactions, and all-cause mortality [19,20]. Furthermore, medication effects are dependent on patient compliance and are of benefit to patients only for the duration of drug therapy, which requires lifelong medication adherence. Metabolic surgery, in comparison, results in marked lifelong physiological changes. Decreasing the patient’s pill burden would also have an impact on the financial burden for both the patient and the health service [21]. 

There is a suggestion from previous studies that tight preoperative glycaemic control is associated with improved rates of diabetes remission [8,22,23]. There remains, however, a scarcity of information on the optimisation of preoperative HbA1c and postoperative weight loss. In this study, there were multiple potential reasons why glycaemic control and weight loss were not maintained post-surgery. In the preoperative phase, the patient’s lifestyle was likely influenced by the education program, which aimed to aid in the optimisation of glycaemic control and weight loss. In addition, the increased use of pharmacotherapy around this period may have aided improvements in HbA1c. These interventions are often regarded by both patients and health professionals as short-term measures working primarily towards the goal of surgery and do not appear to translate into sustainable behaviours associated with long-term weight maintenance following metabolic surgery. 

In this study, 48 (69.6%) patients achieved an improvement in glycaemic control preoperatively (Table 1). The length of follow-up after surgery varies. The NHS recommends two years of follow-up, and the American Society for Metabolic and Bariatric Surgery (ASMBS) and European guidelines do not specify the length of time [24-26]. The European guidelines do, however, recommend ‘lifelong multidisciplinary management’ [26]. Intensive engagement with a multidisciplinary team of healthcare professionals may be more effective if initiated at an earlier stage in the patient’s surgical journey to allow adequate time to establish an individualised preoperative diabetic care plan and permit room for trial and error to find the best strategy for the patient to achieve glucose optimisation and weight loss prior to surgery. Given that long-term relapse of diabetes remission has been shown to occur, it may be prudent to establish a strategy which outlines longer-term patient engagement with the multidisciplinary bariatric team. Evidence of weight regain or diabetes recurrence could, therefore, be identified and addressed at an earlier stage, and interventions could be undertaken to prevent further deterioration [27]. 

This study is limited by the retrospective nature, the small number of patients, which limited the statistical power of the data and possibly selection bias given that patients with insufficient data at certain time points were excluded from the study. It could be that these patients were less likely to engage with the preoperative and postoperative process. Covariate analysis was not undertaken in this study, which acts as a further limitation. Further prospective studies are required to investigate the impact of glucose optimisation on short-term and long-term outcomes following metabolic surgery. 

## Conclusions

In conclusion, this study demonstrated that the mean HbA1c five-year post-surgery was equivalent to that at the time of surgery (6.6%) (Table 2). However, a significant decrease was seen in glucose-lowering medication use five-year post-surgery (p < 0.001), highlighting the potent long-term effects of metabolic surgery on glycaemic control independent of medication use. The study also demonstrated that preoperative alterations in glycaemic control were not correlated with short or long-term weight maintenance or HbA1c in this cohort of patients with T2DM undergoing metabolic surgery. The small sample size and lack of covariate analysis may affect the generalisability of the results, and therefore, larger studies with covariate analysis are required to further assess the impact of preoperative changes in glycaemic control on postoperative weight loss and glycaemic control.
